# Introduction to the *RSC Advances* Emerging Investigators Series 2025

**DOI:** 10.1039/d6ra90129h

**Published:** 2026-10-27

**Authors:** Fabienne Dumoulin, Shirley Nakagaki

**Affiliations:** a Acibadem Mehmet Ali Aydinlar University Türkiye; b Universidade Federal do Paraná Brazil

## Abstract

Professor Fabienne Dumoulin and Professor Shirley Nakagaki are delighted to introduce the *RSC Advances* Emerging Investigators Series, which highlights some of the very best work of early career researchers.
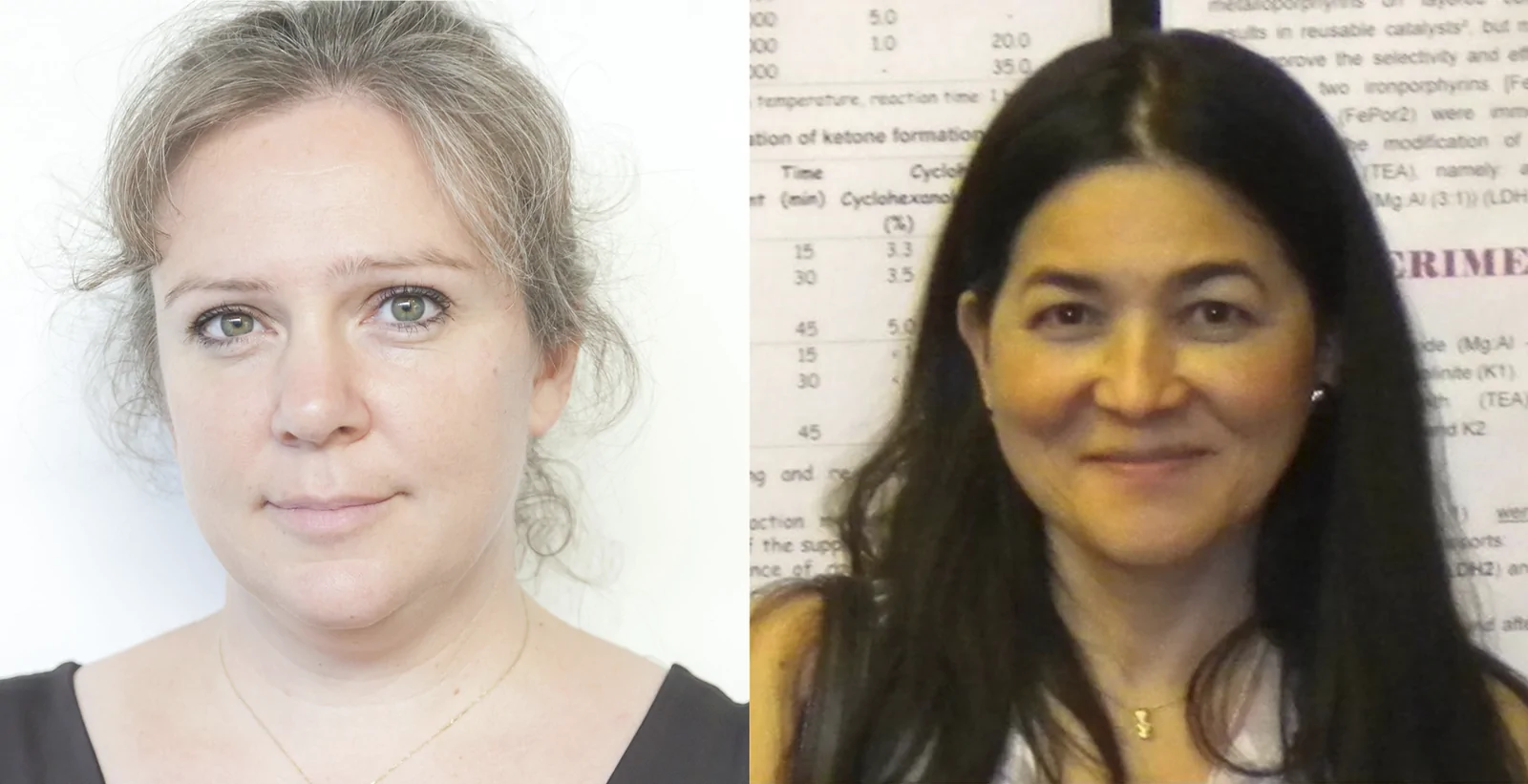

We are proud and delighted to present the 2025 edition of the *RSC Advances* Emerging Investigators Series.

Since the inaugural edition back in 2021, *RSC Advances* are happy to promote works from talented emerging researchers who have recently established their own academic research group. It is for our journal, an honor and a pleasure to be selected for their first publications as independent PIs, and we feel committed to promote them as much as possible.

Unlike previous editions, authors this year had the opportunity to be nominated by members of our Editorial Board or Associate Editors or could self-nominate themselves. Our aim with this change was to have the largest possible number of participants! As a result, we received over 100 applications for this edition; as always, and given the quality of the nominations, it was difficult to choose amongst all the papers eligible to appear in this series. We would therefore like to begin by congratulating everyone who submitted work for the 2025 edition, representing diverse fields of chemical science and countries across every continent.

Compared to previous years, this year was however particularly special, as the world of academic publishing is experiencing a continuously evolving revolution due to the development of generative artificial intelligence. This phenomenon is deeply affecting the entire publishing process, from the science itself to the preparation of manuscripts, their editorial handling and the peer-reviewing process. It is now well-known that this has directly contributed to increasing the numbers of submissions and modified the way science is presented. In this context, *RSC Advances* and the whole Royal Society of Chemistry is establishing and continuously updating rules, while retaining their standards of ethical use, transparency, governance and accountability (Guiding principles for artificial intelligence).

We are extremely pleased that this 2025 issue is particularly inclusive and diverse, thereby reflecting the Royal Society of Chemistry’s ambitious Inclusion and Diversity strategy to 2030 (our inclusion and diversity strategy to 2030) without any compromises on the quality and scientific impact of the selected papers. The diversity also extends to the science, which is very exciting and reflects the various categories of our journal.

There are several papers with a crucial important environmental focus, highlighting how chemistry has a role to play in the current context of climate and biodiversity crises which proved, this summer more than ever, to be needing contributions from all scientific disciplines. Toxic pollutant removal,^1^ including toxic organic dyes^2^ and hydrometallurgical metal recovery^3^ have been tackled in several of the selected papers, as well as pollutant detection,^4,5^ lead and fluoride filtration,^6^ materials for biodiesel production,^7^ as well as many other examples. With a green chemistry approach, the metal-free organic transformations mediated by potassium persulfate^8^ have been investigated. Glyphosate sensors working in water^9^ are another contribution to this environmental field.

Several papers deal with molecules or materials designed for energy applications. The state of the art in the field of 2D materials for energy storage^10^ is presented, the role of machine learning in next-generation AEM fuel cells^11^ have been detailed. The potential of plasmonic nanoparticles to boost low-current perovskite LEDs^12^ is another work in this category.

Biomedical topics also have a big place in our selection, the main focus being cancer and antimicrobial agents and materials. Reports of hydrogels with tailored properties, such as fluorescence^13^ or enhanced mechanical and mineralization performance^14^ have raised our interest. Advanced materials such as carbon nanodots as theranostics agents in cancer^15^ and nanomedicines to overcome cancer resistance^16^ have been reviewed. Docking studies of isatin hybrids with therapeutic potential,^17^ and of antiproliferative cyanoacrylamides,^18^ demonstrate the role of such studies to investigate potential drugs. Furthermore, the mechanism of action of boron-dependent antibiotics has shown to entail synergistic binding.^19^ In a different antimicrobial strategy, photoswitchable glycomimetic ligands of *Pseudomonas aeruginosa* LecB have been reported.^20^

Less common synthetic methods are also reported, in this 2025 edition, such as mechanochemistry used for molybdene complexes^21^ or the use of montmorillonite K10 for the preparation alkyl levulinates.^22^

Finally, more fundamental works have their place in this issue, highlighting the interest and impact of basic science. This is important to point out, as more and more funding bodies request applications and immediate impact in proposals, while we should not forget the importance of pure fundamental studies. The effect of a polymeric layer on jetting dynamics induced by bursting bubbles,^23^ a systematic comparison of density functional methods to study spin crossover complexes^24^ and a comparative work assessing the effect of different macroinitiators of ABA triblock copolymers,^25^ are the examples we chose to include in this issue.

We reiterate that, given the large number of high-quality papers submitted for this edition, selecting the works to comprise the 2025 issue was a herculean task for the editors. Nevertheless, the process filled us with satisfaction and joy, as we know that science – and chemical science in particular – is yielding results and charting new paths across various countries and continents. Congratulations to everyone, and enjoy reading!
